# Glial activation and nociceptive neuropeptide elevation associated with the development of chronic post-traumatic headache following repetitive blast exposure

**DOI:** 10.1016/j.ynpai.2024.100178

**Published:** 2024-12-27

**Authors:** Amirah Wright, Susan F. Murphy, Pamela J. VandeVord

**Affiliations:** a Virginia Polytechnic Institute and State University. Department of Biomedical Engineering, 325 Stranger St., Blacksburg, VA 24060, United States; b Salem Veterans Affairs Medical Center, 1970 Roanoke Blvd, Salem, VA, 24153, United States

## Abstract

Chronic headaches and pain are prevalent in those who are exposure to blast events, yet there is a gap in fundamental data that identifies the pathological mechanism for the chronification of pain. Blast-related post-traumatic headaches (PTH) are understudied and chronic pain behaviors in preclinical models can be vital to help elucidate PTH mechanisms. The descending pain modulatory system controls pain perception and involves specific brain regions such as the cortex, thalamus, pons, and medulla. In this study, male rats were exposed to repeated blast events to induce traumatic brain injury (bTBI) and subsequently assessed for the development of PTH by testing for chronic pain behaviors and examining the neuropathology of the descending pain pathway. The results demonstrated that facial hypersensitivity developed as early as week two following bTBI and persisted throughout the study (12 weeks). Depressive-like behaviors were observed at 12 weeks following bTBI, and these behaviors were associated with neuropathologies such as microglia ramification and neuropeptide elevation (Calcitonin Gene-Related Peptide, CGRP; Substance P, SP). Overall, these findings support the hypothesis that bTBI causes the activation of microglia and elevation of neuropeptides, which contribute to the development of chronic PTH behaviors.

## Highlights

• Repeated bTBI leads to chronic post-traumatic headache associated behaviors.
• PTH after bTBI linked to microglial activation and decreased astrocyte reactivity.
• Elevated nociceptive neuropeptides seen after repeated bTBI at a chronic timepoint.
• This suggests glial cells and nociceptive neuropeptides work in tangent in PTH pathology after bTBI.

## 1 Introduction

Chronic headaches and traumatic brain injury (TBI) are common co-morbidities in individuals who have experienced concussive events. Chronic headaches/migraines occur in 1–4 % of civilians (Murphy and Hameed, 2024) but were elevated in > 20 % of the post-9/11 combat Veteran population (Williams, 2020). Post-traumatic headaches (PTH) make up 4 % of all headache disorders (Ashina et al., 2020). The most common TBI mechanism in the Veteran population is exposure to an explosive blast wave (Wilk et al., 2010, Masel et al., 2012, Ratliff et al., 2020). While most cases are considered mild (mTBI), they are reported to lead to the development of PTH, especially when repeated exposures are involved (Walker et al., 2023). There is a higher prevalence of headache symptoms among combat Veterans. Of the risk factors for developing PTH (e.g., injury mechanism, severity, sex), blast-related TBI (bTBI) was the leading factor and was uniquely associated with elevated PTH risk (Walker et al., 2023).

PTH impacts a person’s overall health and quality of life, often leading to psychological and cognitive deficits (Tracey and Mantyh, 2007, Monteith, 2018). PTH patients also suffer from secondary symptoms, most commonly changes in pain sensitivity (Bernstein and Burstein, 2012). Patients who suffer from TBI and PTH experience thermal hypoalgesia and mechanical hyperalgesia in the facial region (Defrin et al., 2012, Defrin et al., 2015). The underlying mechanisms attributed to these symptoms involve both the central and peripheral nervous systems (Tracey, 2007, Bree and Levy, 2018). Researchers have studied the connection between the locations where patients feel pain and the underlying nervous system pathology that contributes to that particular symptomology (Boyer et al., 2014, Defrin, 2014, Defrin et al., 2015). Clinical imaging studies showed that input from the ascending track activated the descending pain pathway, causing neurons within the pain system to become active and hypersensitive (Valet et al., 2004, Tracey, 2007, Boyer et al., 2014, Strigo et al., 2014). Researchers suggest that the activation of the neurons may be due to the frontal brain sending signals through neurons within the trigeminovascular system to release pain-provoking substances, e.g., Substance P (SP) and Calcitonin gene-related-peptide (CGRP) that then bind to their respective receptors on cells adjacent to neurons (Elliott et al., 2012, Assas et al., 2014).

The neuropathology mechanism has been linked to various injury paradigms and includes neuroinflammation, glial reactivity, and neuronal dysfunction (Cernak and Noble-Haeusslein, 2010, Mayer et al., 2013, Siedhoff et al., 2021). Multiple signaling pathways likely contribute to the progression of pain from acute to chronic. Previous studies show that SP and CGRP are released from sensory neurons, contributing to the development and maintenance of chronic headaches/migraines (Greco et al., 2008, Assas et al., 2014). These neuropeptides can then bind to and activate resident central nervous system (CNS) cells (Burmeister et al., 2017, Carniglia et al., 2017, Afroz et al., 2020). Glial cells, specifically astrocytes and microglia, have been a focus of studies due to their contribution to the inflammatory immune response. These resident brain cells are known to drive the transition from acute pain to chronic if inflammatory signaling is dysregulated as a result of TBI (Watkins et al., 2001, McMahon et al., 2005, Mayer et al., 2013). Infiltrating and resident immune cells causes the neuropeptides to signal nociceptors through the release of mediators that act on the terminals of the nociceptors, whichactivates a feedback-loop process and then causes surrounding glial cells and neurons to maintain pain sensitization (Milligan and Watkins, 2009, Ji et al., 2016, Baral et al., 2019, Gregus et al., 2021).

Clinical studies have identified a connection between bTBI and PTH development, however few preclinical studies have tried to identify pathological changes that lead to the development of PTH after bTBI, especially on a chronic scale. This study aimed to determine whether bTBI leads to chronic PTH using a military-relevant preclinical model. Four key brain regions that play a role in pain response were examined. We hypothesized that blast exposure leads to PTH pathology in these regions that correlate with facial pain-behavioral phenotypes.

## 2 Methods

### 2.1 Blast Methods and animal Description

All experimental protocols were approved and performed in accordance with the Virginia Tech Institutional Animal Care and Use Committee guidelines and regulations. Male Sprague Dawley rats (Envigo, Dublin, VA) were housed in pairs following a 12-hour light–dark cycle, with food and water administered *ad libitum*. The animals were allowed to acclimate for one week before commencing experiments. They were 10 weeks old and weighed 295.65 ± 5.24 g at the time of injury.

**Fig. 1:**
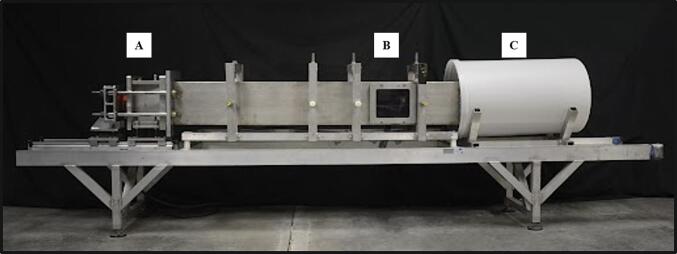
The VT Advanced Blast Simulator (ABS) generates a blast wave to induce bTBI in rats. The ABS is pressurized via compressed helium gas within the driver section **(A)** until a membrane bursts. The wave propagates over the test section **(B)** and dissipates in the end wave eliminator **(C)**.

Blast waves were generated using a custom Advanced Blast Simulator (ABS). The ABS (Stumptown Research & Development, Black Mountain, NC, USA) consists of three sections that create, expel, and dissipate a blast wave (Fig. 1). The blast wave was created by a helium-driven rupture of a vinyl membrane and dissipated in an end wave eliminator. The result was a single peak overpressure representing a free-field blast wave as seen in real-life combat scenarios (VandeVord et al., 2016, Nelson et al., 2024). The pressure measurements were collected using a Dash 8HF data (AstroMed) acquisition system. Pressure data was analyzed using a custom MATLAB script to calculate impulse, peak pressure, duration of the positive phases, and rise time. A detailed protocol can be found at protocols.io (VandeVord, 2023).

Before the blast procedure, animals were anesthetized via 4 % isoflurane delivered through a surgical vaporizer connected to an induction chamber. The animals were then positioned in a taut mesh sling facing the oncoming blast wave, thus a full-body exposure. Each animal received three blast insults (19.048 psi ± 0.272) separated by one hour (3 x 1 hr; n = 10) to mimic the pressures and repetition that military personnel may experience in combat. The sham group underwent the same procedures except for the blast exposure (n = 10).

### 2.2 Facial mechanical hyperalgesia

The animals underwent weekly mechanical hyperalgesia testing for 12 weeks post-blast, starting at week two. The test was performed by wrapping the animals in a towel as a restraint and then using calibrated von Frey filaments to apply a stimulus for 1–2 s to the periorbital region of the face. The filaments ranged between 10–300 g of force, starting with the lowest filament and then increasing in force. The filament that caused a physical and/or vocal reaction from the animal was recorded as a pain response. A detailed protocol can be found at protocols.io (Wright et al., 2024).

### 2.3 Open field test (OFT)

The OFT measured overall activity levels and anxiety-like behaviors that may develop with time (Doga Vuralli, 2019). The test was conducted one week post-blast and then every four weeks for 12 weeks. Briefly, the animal was placed in an 80 cm^2^ arena in an isolated room (no investigators present) for five minutes. An overhead camera recorded the behavior, and the captured video was analyzed using EthoVision XT tracking software (Noldus Information Technology, Leesburg, VA, USA). The videos were verified for any tracking discrepancies. The specific parameters quantified were mean velocity, time spent along the wall (thigmotaxis), time in the center area of the arena, frequency of visits into the wall, frequency of visits to the center area, and time it took to enter (latency to first) the center area of the arena.

### 2.4 Elevated plus maze (EPM)

EPM was performed 12 weeks following repeated bTBI. The test measures anxiety-like behaviors based on the desire to explore new environments and/or avoid potentially dangerous areas (Dickerson et al., 2021). The behavior apparatus is a plus-shaped maze consisting of two enclosed arms and two open arms intersected with a center square. The test was performed in an isolated room (no investigators present) with an overhead camera recording the behavior. Animals were placed into the center of the maze facing towards the same open arm and recorded for five minutes. Video files captured were analyzed using EthoVision XT tracking software, and captured videos were verified for any tracking discrepancies. The specific parameters quantified were mean velocity, time spent within each zone of the arena, frequency of visits into each zone, and time it took to enter (latency to first) the closed arms, open arms, and center of the arena.

### 2.5 Triple-Chamber sociability and social novelty test

The triple-chamber test was performed 12 weeks following repeated bTBI (Dickerson et al., 2021). The test consisted of three 10-minute trials. In the first trial, the test rat had a habituation period where all the chambers were empty and the doors were open. In the second trial, the sociability test, the test rat was placed in the center chamber with the doors in place, and a stranger rat was placed in a cage enclosed in one of the chambers. The opposite chamber contained an identical, empty cage. The cage had clear bars that allowed for nose contact between the bars. The test rat was allowed to explore the entire arena for 10 min. In the third trial, the social novelty test, the test rat was again placed in the center chamber, and the stranger rat remained in the cage while a novel rat was placed in a cage in the third chamber with the same clear bars to allow for nose contact. The test rat was again allowed to explore the entire arena for 10 min. The test was performed in an isolated room (no investigators present) with an overhead camera recording the behavior. Video files captured were analyzed using EthoVision XT tracking software. They were then verified for any tracking discrepancies. The second and third trials were analyzed as separate tests. For each test, the following parameters were quantified: mean velocity, distance traveled, time spent in the sniff zones (designated zones around the stranger animals’ cages), and chambers, the number of times the animal entered the sniff zones and chambers, and the time it took to enter (latency to first) the sniff zones and chambers the first time.

### 2.6 Immunohistochemistry (IHC)

Twelve weeks post-blast exposure, animals were anesthetized with 5 % isoflurane and euthanized by exsanguination, then transcardially perfused with 0.9 % saline, followed by 4 % PFA. Brains were extracted and post-fixed for 24 h, followed by a phosphate buffer saline (PBS) rinse, then dehydrated using a 30 % sucrose solution. The brains were then embedded in an optimal cutting temperature medium and frozen at −80 °C. Sagittal sections were cut (40 µM) and stored in PBS at 4 °C. The regions of interest (ROI) included the cortex, thalamus, pons, and medulla. The sections were stained with the following antibodies (Table 1): Glial Fibrillary Acidic Protein (GFAP), Ionized Calcium-Binding Adaptor Molecule 1 (IBA-1), Calcitonin Gene-Related Peptide (CGRP), and Substance P (SP). Sections were rinsed with PBS and PBS containing 0.03 % Triton X and incubated in a blocking buffer (2 % bovine serum albumin (BSA)) for one hour. Sections were then incubated in primary antibodies diluted with blocking buffer for 16–18 h at 4 °C. Next, they were washed with PBS and then incubated with their secondary antibodies diluted with blocking buffer: CGRP (Alexa Fluor 647), SP (Alexa Fluor 647), and GFAP (Alexa Fluor 488) were pre-conjugated with their respective fluorochrome. After incubation, samples were washed and then incubated in DAPI for 10 min, washed with PBS, mounted on slides, dried, and coverslipped with Antifade Gold Mountant. The slides were examined and imaged on a Zeiss fluorescence microscope at 20x magnification.

**Table 1:** Antibodies used for histological analyses.

Antibody	Vendor	Cat #	Dilution
SP	Bioss Antibodies	bs-0065R-BF594	1:400
CGRP	Bioss Antibodies	bs-18210R-BF647	1:400
GFAP	Cell Signaling Technology	3670S	1:500
IBA-1	Biocare Medical	CP290B	1:300
Anti-Rabbit IgG	Invitrogen	A10040	1:500

### 2.7 Statistical analysis

A student’s *t*-test was used to compare groups. A one-way ANOVA with Tukey’s post-hoc test was used to correct for multiple comparisons when appropriate. A two-way ANOVA with Tukey’s post-hoc test was used to analyze the differences in time or region between the groups and their effect on the observed outcomes. Outliers were identified and excluded from further analysis based on the ROUT method. The Brown-Forsythe and Shapiro-Wilk tests were used to verify assumptions of homoscedasticity and normality, respectively. If the data failed these assumptions, Welch’s correction *t*-test or the Kruskal-Wallis non-parametric test was performed. Data was deemed significant if p < 0.05 and trending when p < 0.1. Data was normalized to the shams, representing the mean ± standard error of the mean (SEM). GraphPad Prism software (GraphPad Software, La Jolla, CA) was used for all statistical analyses.

## 3 Results

### 3.1 Blast Event

**Fig. 2:**
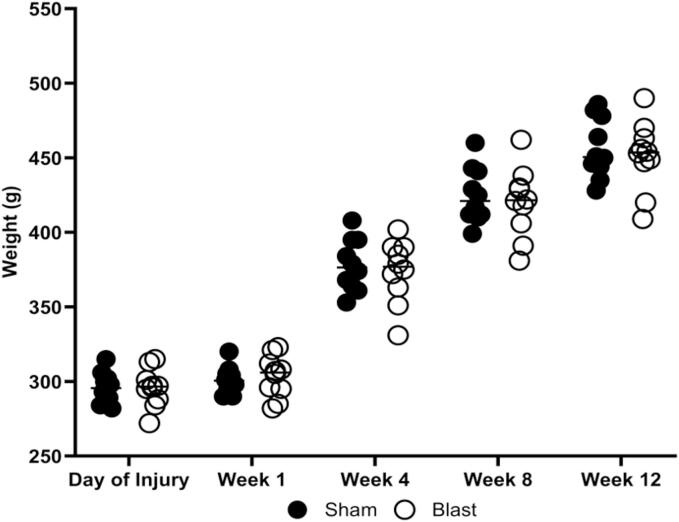
Change in weight over 12 weeks in the blast and sham groups. Data is represented as Mean ± SEM.

Blast animals were exposed to three blasts separated by one hour each. Blastwave characteristics are shown in Table 2. The animals’ weights were monitored every four weeks until they were euthanized at 12 weeks post-blast. A consistent and healthy weight gain was maintained throughout the study, and there were no significant differences in weight between the injured and sham animals (Fig. 2).

**Table 2:** Blast wave characteristics.

Blast Interval	Peak Positive Pressure (psi)	Positive Duration (ms)	Rise Time (ms)
3 x 1 hr	19.02 ± 0.03	2.29 ± 0.01	0.11 ± 0.003

### 3.2 Blast TBI-induced facial hyperalgesia

**Fig. 3:**
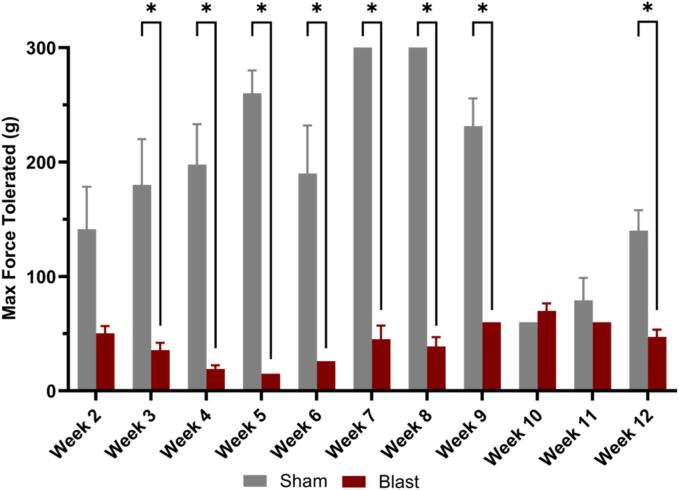
Facial hyperalgesia was assessed using the von Frey test. Periorbital pressure measurements (g) were taken at each weekly time point post-injury. Facial sensitivity was significantly increased in bTBI animals compared to sham at two weeks post-injury, persisting until 12 weeks post-injury compared to sham animals. Two-way ANOVA post-hoc test. Data is represented as Mean ± SEM, *p < 0.05.

Previous studies have shown that humans have altered pain sensitivity, including a decreased pressure pain threshold, after a blast injury, which was also linked to those who experience chronic headaches (Bernstein and Burstein, 2012, Defrin et al., 2015). Von Frey filaments were used as a mechanical test to quantify facial hyperalgesia weekly following blast (Fig. 3). The 2-way ANOVA results revealed a significant reduction in the maximum force tolerated by blast animals compared to sham (p < 0.05). Blast animals showed a significant hypersensitivity to stimuli that started two weeks post-blast and persisted up to the nine-week time point post-blast based on the maximum force tolerated. Statistically, there was no difference between blast and sham in weeks 10 and 11, but at 12 weeks, there was a statistically significant reduction in tolerance in blast animals compared to sham. Importantly, periorbital hypersensitivity that occurred over 12 weeks demonstrated that bTBI led to chronic pain conditions.

### 3.3 Open field test

**Fig. 4:**
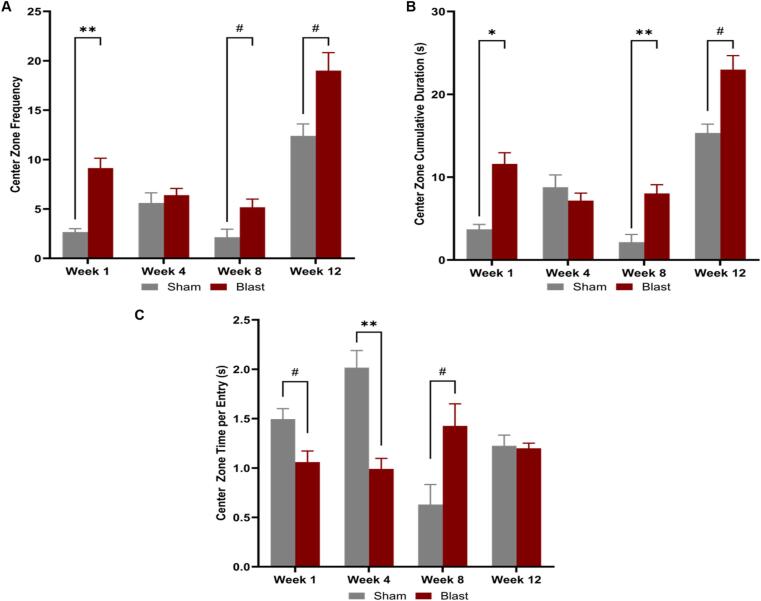
Anxiety-like behaviors were measured using the OFT. At 1-, 8-, and 12 weeks after injury, blast animals exhibited risk-taking behavior with increased entries **(A)** and increased cumulative duration **(B)** in the center of the maze. At 1- and 4 weeks post-injury, the blast group spent significantly less time per entry than sham but more time per entry at 8 weeks **(C)**. Two-way ANOVA with Post-hoc test. Data is represented as Mean ± SEM, ^#^p ≤ 0.1, *p < 0.05, ** p < 0.01.

The open field test was used to examine differences in anxiety-like behaviors and was conducted at 4-week intervals, starting 1-week post-blast. A 2-way ANOVA was conducted to analyze exploration of the center zone of the arena based on the factors of injury group and time-point. Blast animals showed increased risk-taking behaviors at 1, 8, and 12 weeks post-injury, entering the center more frequently (Fig. 4A) and spending more time in the center (Fig. 4B) compared to the sham group. However, at 1- and 4 weeks after injury, the blast animals spent less time per entry (Fig. 4C) than sham, but at 8 weeks, they spent more time than the sham group. This behavior change illustrates the risk-taking tendencies of the injured animals at that time point. The results of the 2-way ANOVA indicated an interaction between the injury group and time-point, suggesting that both the injury (bTBI) and the time post-blast influenced center zone exploration during the open field test.

### 3.4 Elevated plus maze

**Fig. 5:**
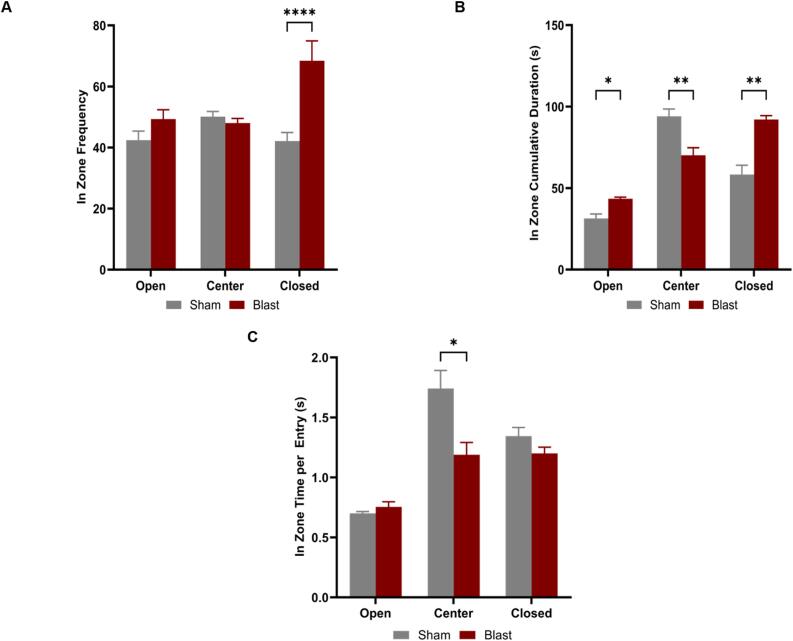
Elevated Plus Maze was used as another measure of anxiety-like behaviors. At 12 weeks, blast animals frequented the closed arms of the maze significantly more times than sham **(A)**. They also spent more time in the closed and open arms but less time in the center of the maze compared to sham **(B)**. The sham group spent more time per entry into the center compared to injured animals **(C)**. One-way ANOVA with Tukey’s post-test. Data is represented as Mean ± SEM, *p < 0.05, ** p < 0.01, ****p < 0.001.

At 12 weeks post-blast, the animals underwent an EPM assessment for anxiety-like behaviors. The blast group entered the closed arms of the maze significantly more times than the sham group (p = 0.0111) (Fig. 5A). They spent significantly more time (Fig. 5B) in the closed (p = 0.0011) and open arms (p = 0.0101) compared to sham. Interestingly, blast animals spent less time (Fig. 5B) in the center (p = 0.0065) and significantly less time per entry (Fig. 5C) to the center (p = 0.0235) than sham.

### 3.5 Blast animals showed sociability deficits

**Fig. 6:**
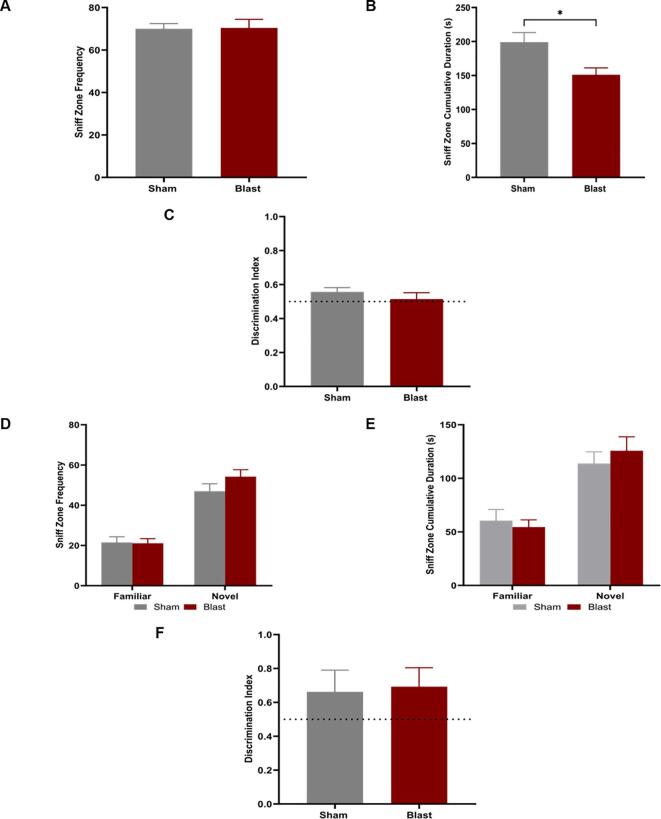
Triple Chamber Test for depressive-like and anxiety-like behavior. The triple chamber test was used to determine the time spent socializing with stranger animals. T-tests indicated that blast animals had significant depressive-like behaviors compared to sham. In the sociability phase of the test, **(A)** blast animals made significantly fewer entries to the sniff zone of the stranger rat than sham and **(B)** the blast animals spent significantly less time in the sniff zone with the stranger rat than sham. **(C)** The discrimination index for chamber preference was compared between groups. There were no significant differences in indices. In the Social Novelty phase of the test, **(D)** there was no significant difference between blast and sham in the frequency of entries to both sniff zones **(E)** There was no difference between blast and sham groups in the amount of time spent with either animal. **(F)** The discrimination index for sniff zone preference was compared between groups. There were no significant differences in indices. Student’s *t*-test. Data is represented as Mean ± SEM, *p < 0.05.

At 12 weeks following bTBI, the triple chamber sociability test was used to measure social deficits, commonly associated with depressive symptoms (Elmer and Stadtfeld, 2020). The blast animals entered the stranger sniff zone significantly fewer times than the sham animals (p < 0.05; Fig. 6A). Blast animals spent significantly less time within the sniff zone around the stranger rat than sham (Fig. 6B), but there was no difference in time spent within the chamber that contained the stranger rat compared to sham rats (Fig. 6C).

There was no difference in social novelty behavior between blast and sham (Fig. 6D-F). For all animals, there were significantly more entries to the sniff zone of the novel animal (p < 0.0001) compared to the now-familiar animal, and they spent significantly more time around the novel animal compared to the now-familiar animal (p < 0.001), suggesting that the injured animals have no deficits in social memory.

### 3.6 Immunohistochemistry

#### 3.6.1 Microglia morphology changed in response to blast injury

**Fig. 7:**
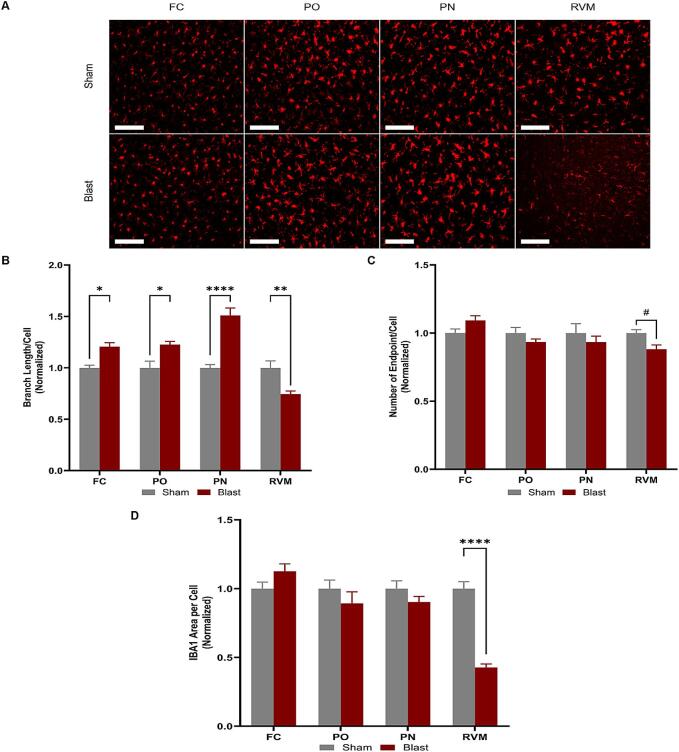
Microglia reactivity was measured through the morphological changes within each region and group. **(A)** Representative images depicting the morphological changes within the regions of interest. **(B)** There were significant differences in summed branch length per cell between the blast and sham groups across the four regions. **(C)** There was a decreasing trend in the number of endpoints in the RVM in blast animals but no significance in any other ROI. **(D)** There was a significant decrease in area per cell of IBA-1 in the RVM in blast animals but no significant differences across the other three ROIs. Frontal cortex (FC), posterior nucleus (PO) in the thalamus, pontine nucleus (PN), and the rostral ventromedial medulla (RVM). One-way ANOVA. Data is represented as Mean ± SEM, ^#^p ≤ 0.1, *p < 0.05, **p < 0.01, ****p < 0.001. All scale bars, 4.66 µm.

Microglia release mediators that sensitize nociceptors that, in turn, trigger glial cells and neurons to maintain a hyperalgesia state (McMahon et al., 2005, Ji et al., 2013). In addition, research has shown that microglia undergo morphological changes in response to TBI (Eggen et al., 2013, Morrison et al., 2017). To analyze microglial morphology, tissue from 12 weeks post-bTBI was stained with an IBA-1 antibody, imaged, and subsequently analyzed in ImageJ using the method established by Young and Morrison (2018) (Fig. 7A). A two-way ANOVA revealed that both the injury group and brain region significantly affected morphology and IBA-1 expression. Significant differences were observed in branch length per cell between blast and sham animals across all four ROIs (interaction: p = 0.0270) (Fig. 6B). However, there were regional differences in whether the blast group had an increased (FC, PO, and PN) or decreased (RVM) branch length compared to sham (Fig. 7B). The RVM demonstrated a noticeable decrease in the number of endpoints per cell (Fig. 7C) compared to the other regions. However, there were no significant differences in endpoints per cell between the blast and sham groups; this suggests that microglia may either become de-ramified or hyper-ramified, depending on the specific region. The area per cell (Fig. 7D; interaction: p = 0.0001) calculated in the ROIs revealed a significant decrease within the RVM. The combined decrease in all three measurements suggests the microglia within the RVM are more de-ramified and more “active” compared to the other regions housing “resting” microglia.

#### 3.6.2 Astrocyte expression differences seen at the chronic time point

**Fig. 8:**
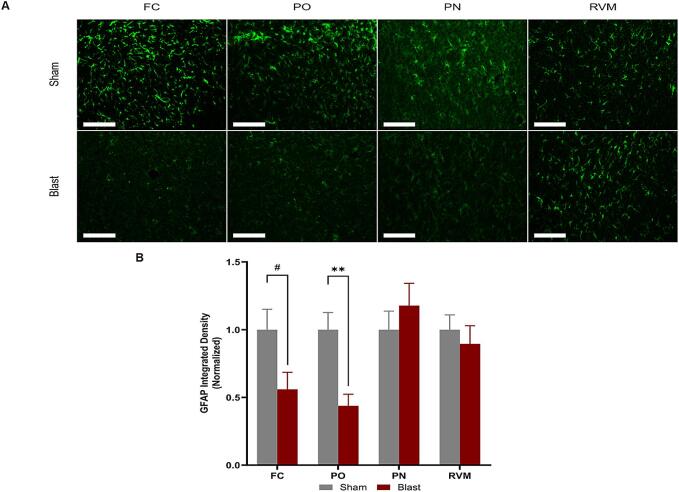
GFAP astrocyte activity was assessed through immunohistochemistry staining for GFAP. **(A)** Representative images of GFAP within the regions of interest. **(B)** GFAP expression within the FC (p = 0.1013) and PO (p = 0.0018) regions was reduced in the blast animals compared to sham. Frontal cortex (FC), posterior nucleus (PO) in the thalamus, pontine nucleus (PN), and the rostral ventromedial medulla (RVM). One-way ANOVA Data is represented as Mean ± SEM, *p < 0.05, ^#^p ≤ 0.1. All scale bars, 4.66 µm.

Gliosis is a key mechanism for driving pain development through neuroinflammatory processes (Watkins et al., 2001). There are multiple ways of assessing astrocytic activity, one of which is measuring the expression of GFAP, the most commonly used astrocytic marker (Fig. 8A). At 12 weeks, astrocyte expression of GFAP was significantly decreased within the frontal cortex and posterior nucleus regions of injured animals compared to sham (Fig. 8B).

#### 3.6.3 Regional differences in SP and CGRP expression

**Fig. 9:**
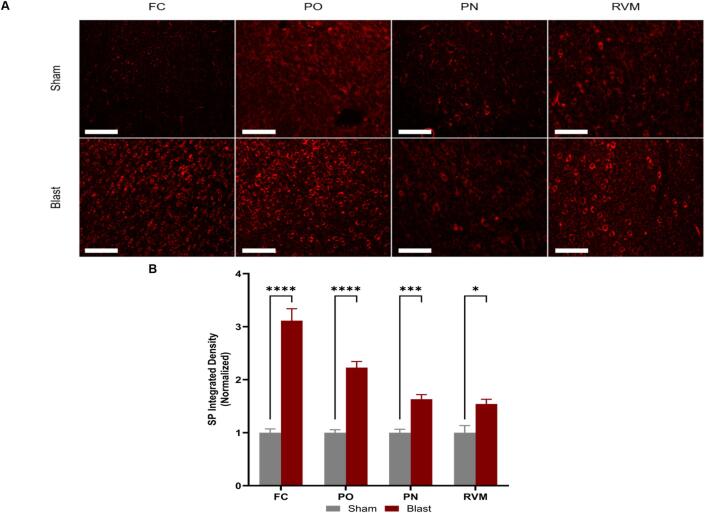
Substance P **(A)** Representative images showing expression of Substance P within the regions of interest. **(B)** Substance P increased significantly in all ROIs of blast animals compared to sham. Frontal cortex (FC), posterior nucleus (PO) in the thalamus, pontine nucleus (PN), and the rostral ventromedial medulla (RVM). One-way ANOVA Data is represented as Mean ± SEM, *p < 0.05, *** p < 0.005, **** p < 0.001. All scale bars, 4.66 µm.

**Fig. 10:**
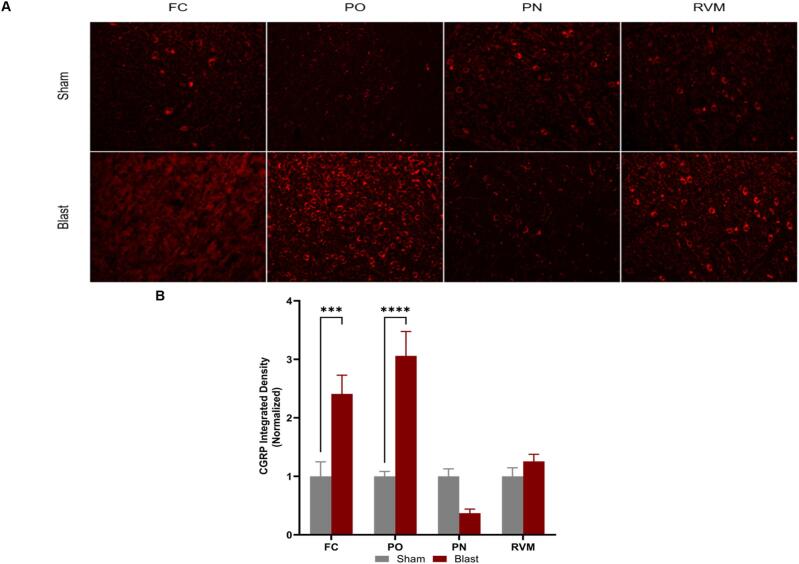
Calcitonin gene-related neuropeptide activity (CGRP). **(A)** Representative images of CGRP within the regions of interest. **(B)** Integrated density. There was a significant increase in CGRP expression in blast animals in the FC and PO. Frontal cortex (FC), posterior nucleus (PO) in the thalamus, pontine nucleus (PN), and the rostral ventromedial medulla (RVM). One-way ANOVA data is represented as Mean ± SEM, ***p < 0.005, **** p < 0.001. All scale bars, 4.66 µm.

The neuropeptides SP (Fig. 9A) and CGRP (Fig. 10A) were selected as pain-associated biomarkers since they play critical roles in the secondary injury process concerning neuroinflammation (Dara Bree, 2018). Their release from sensory nerve fibers is also necessary for the development and chronification of pain (Young and Morrison, 2018). A two-way ANOVA showed that the interaction between the injury group and region of interest had a significant effect on the expression level of both SP (Fig. 9B; interaction: p = 0.0001) and CGRP (Fig. 10B; interaction: p = 0.0001) as they were elevated at three months post-blast. SP specifically showed increased expression within all four ROIs, while CGRP was increased in the frontal cortex and posterior nucleus.

## 4 Discussion

This study was the first to demonstrate that repeated bTBI was associated with the development of hypersensitivity, which progressed to the chronic phases of bTBI. Further, results were associated with glial reactivity and an increased expression of CGRP and SP, two neuropeptides previously shown to be related to chronic headache outcomes and secondary neuropathology after TBI (hubin Duan, chunyan Hao, shuzhen Li. , 2009, Lorente, 2015, Vink et al., 2017, Bree and Levy, 2018, Navratilova et al., 2019, Edvinsson, 2021). These results validate the debate that exposure to blast leads to long-term sequelae, such as chronic PTH reported by patients suffering from bTBI (Tracey and Mantyh, 2007, Monteith, 2018, Meltzer and Juengst, 2021).

While limited preclinical studies have investigated the link between bTBI and chronic pain development (Studlack et al., 2018, Uddin et al., 2019, Wattiez et al., 2021), it was reported that animals experience acute and chronic facial hypersensitivity following TBI but experimental models vary in animal species and injury protocols. Wattiez et al. showed that a single and repeated bTBI could induce periorbital and plantar tactile hypersensitivity up to 63 days after injury in mice (Wattiez et al., 2021). Using a direct cranial blast injury model, Studlack et al. found that rats exposed to a bTBI experienced significant orofacial pain up to four weeks post-injury but no chronic anxiety-like behaviors (Studlack et al., 2018). This study found that a bTBI resulted in similar increased facial hypersensitivity that persisted until the 12-week endpoint (with some naturally expected fluctuation). Of the previous bTBI pain studies, only two looked into additional PTH symptoms, such as anxiety, at four weeks post-injury and found conflicting results (Studlack et al., 2018, Uddin et al., 2019). However, the current study found significant changes in anxiety-like behaviors across several time-points over 12 weeks in the injury paradigm. Additionally, the previous studies did little to no examination of the underlying molecular pathology contributing to PTH-associated behaviors such as facial sensitivity, depression, and anxiety. Therefore, there was a need to delve deeper into the neuropathology, such as the role of glial cells and neuropeptides across the brain, causing chronic PTH.

Previous TBI studies have examined glial cell activity (Elliott et al., 2012, Studlack et al., 2018, Uddin et al., 2019) as an important contributor to inflammatory processes. The current study measured microglia morphology and astrocyte reactivity within the cortex, thalamus, pons, and medulla. These regions of interest have been shown to play critical roles in pain pathways (Tracey, 2007, Tracey and Mantyh, 2007, Bernstein and Burstein, 2012). Since the current blast model has demonstrated diffuse brain injury, it was hypothesized that these regions would be susceptible to the injury. At 12 weeks post-injury, significant GFAP-labeled astrocyte expression was observed in the posterior nucleus and frontal cortex but not within the pontine nucleus or rostral ventromedial medulla. This decrease in GFAP was associated with the increase in anxiety-like and depressive-like behaviors seen in the bTBI animals, mainly when this change occurs in the prefrontal cortex, thalamus, and amygdala (Dickerson et al., 2020, Dickerson et al., 2021). There were also chronic alterations in microglia morphology following bTBI. Branch lengths were significantly increased in the blast animals within the frontal cortex, posterior nucleus, and pontine nucleus, but decreased in the rostral ventromedial medulla; this suggests that the microglia within the medulla assumed more of an amoeboid or “activated” state than the other regions examined. However, regions in the brainstem naturally have a low density and ramification of microglia compared to other regions in the brain (Yun-Long Tan, 2020). The other regions show increased branch length; this could indicate a ramified or hyper-ramified morphology. While many studies have called this the “resting state,” mounting evidence shows that these microglia are activated due to their constant dynamic changes (Nimmerjahn et al., 2005, Vidal-Itriago et al., 2022). Two previous studies (Dickerson et al., 2020, Dickerson et al., 2021) using the same 3x1-hour blast injury paradigm analyzed gliosis at 4- and 52 weeks post-blast. At four weeks post-injury, no significant differences in astrocyte expression were found between blast and sham in regions evaluated; however, expression of IBA-1 increased in the central medial nuclei of the thalamus, and there was an increase in microglia expression within the ventrolateral nuclei in the thalamus (Dickerson et al., 2020). At 52 weeks, there were significant decreases in GFAP expression in the motor cortex (Dickerson et al., 2021). This evidence shows how glial reactivity will change over time after blast injury and could contribute to chronic headaches.

Evidence shows that glial cells interact with neuropeptides (CGRP and SP) in different pain disorders (Watkins et al., 2001, Sun et al., 2021). Furthermore, CGRP and SP are two well-known neuropeptides implicated in headache disorders (Greco et al., 2008, Bree and Levy, 2018, Zieglgansberger, 2019, Edvinsson, 2021). In this study, CGRP was increased at 12 weeks post-injury in the frontal cortex and posterior nucleus of the thalamus, coinciding with the increased periorbital hypersensitivity the injured animals experienced at that time point. This could be further evidence that CGRP plays a role in chronic PTH development after blast injury, especially since the thalamus, such as the posterior nucleus, is the site of heightened pain-evoking responses and relays this information to the cortex (Ab Aziz and Ahmad, 2006). However, there were no significant changes in CGRP levels within the PN and RVM, which was not expected as CGRP is released from the trigeminal ganglion to the brainstem trigeminal complex in order to transmit nociceptive information (Goadsby et al., 2009); this could indicate the time-point dependence of the injury that may not be focused on transmitting pain signals but on processing and perceiving said pain signals. SP is known to transmit nociceptive signals to the spine and brainstem but has yet to be extensively observed in the thalamus and cortex (Zieglgansberger, 2019). At 12 weeks, SP increased in expression after bTBI within all regions examined. Only one study has examined the link between SP and bTBI and found that blocking the neuropeptide decreased tau phosphorylation at 24 h and 28 days after injury (Corrigan et al., 2021). These results, in conjunction with observed behavioral outcomes, further exemplify the part these neuropeptides play in PTH symptoms at chronic time points. Our study is a step in determining which markers play critical roles in the chronification of pain after a blast injury and which regions contribute to pain modulation.

Since this study was limited to examining the injury response of male rats, future directions should include studying this injury paradigm in a female preclinical model due to the complexity of TBI itself (McCabe and Tucker, 2020). Few studies have included female animals in preclinical bTBI models. In one such study, differences were observed in the severity of symptoms based on sex, with females showing a greater risk for more severe outcomes (Hubbard et al., 2022). Also, several models of chronic pain demonstrated that female rats use adaptive immune responses while male rats use their innate immune response during injury recovery (Gregus et al., 2021). This knowledge could customize clinical treatment for females, as females have a higher rate of reported chronic PTH than males (Hoffman et al., 2011, Murphy and Hameed, 2024). These findings can lead to new diagnostic tools and therapeutics for clinical use that can aid in helping bTBI patients improve their quality of life.

In conclusion, the data collected from this study indicated that headache and pain-like behaviors (i.e., facial hypersensitivity) are significant in males after bTBI. Risk-taking and depressive-like behaviors were observed following injury, which is a common symptom reported in Veterans with chronic PTH (Defrin, 2014, Phipps et al., 2020, Meltzer and Juengst, 2021) and is consistent with previous results (Dickerson et al., 2021). In addition, the data revealed that altered neuropeptides, CGRP, and SP expression levels drive these pain-like behaviors. However, the correlation between bTBI and chronic pain remains an area that warrants further exploration and study.

## Funding statement

This research was conducted without financial support from any funding agency.

## CRediT authorship contribution statement

**Amirah Wright:** Writing – review & editing, Writing – original draft, Visualization, Validation, Resources, Project administration, Methodology, Formal analysis, Data curation, Conceptualization. **Susan F. Murphy:** Writing – review & editing, Validation, Supervision, Methodology, Investigation, Conceptualization. **Pamela J. VandeVord:** Writing – review & editing, Validation, Supervision, Resources, Project administration, Methodology, Funding acquisition, Conceptualization.

## Declaration of competing interest

The authors declare that they have no known competing financial interests or personal relationships that could have appeared to influence the work reported in this paper.

## Data Availability

Data will be made available on request.
